# A message to Fukushima: nothing to fear but fear itself

**DOI:** 10.1186/s41021-016-0039-7

**Published:** 2016-06-01

**Authors:** Shizuyo Sutou

**Affiliations:** School of Pharmacy, Shujitsu University, 1-6-1 Nishigawara, Naka-ku, Okayama 703-8234 Japan

**Keywords:** Atomic bomb survivors, Cancer risk, Fear to radiation, Hiroshima and Nagasaki, Hormesis, LNT, LSS, Linear no-threshold, Lifespan study

## Abstract

**Introduction:**

The linear no-threshold model (LNT) has been the basis for radiation protection policies worldwide for 60 years. LNT was fabricated without correct data. The lifespan study of Atomic bomb survivors (LSS) has provided fundamental data to support the NLT. In LSS, exposure doses were underestimated and cancer risk was overestimated; LSS data do not support LNT anymore. In light of these findings, radiation levels and cancer risk in Fukushima are reexamined.

**Results:**

Soon after the Fukushima accident, the International Commission on Radiological Protection issued an emergency recommendation that national authorities set reference highest levels in the band of 20–100 mSv and, when the radiation source is under control, reference levels are in the band of 1–20 mSv/y. The Japanese government set the limit dose as low as 1 mSv for the public and stirred up radiophobia, which continues to cause tremendous human, social, and economic losses. Estimated doses in three areas of Fukushima were 0.6–2.3 mSv/y in Tamura City, 1.1–5.5 mSv/y in Kawauchi Village, and 3.8–17 mSv/y in Iitate Village. Since even after acute irradiation, no significant differences are found below 200 mSv for leukemia and below 100 mSv for solid cancers. These data indicate that cancer risk is negligible in Fukushima. Moreover, beneficial effects (lessened cancer incidence) were observed at 400–600 mSv in LSS. Living organisms, which have established efficient defense mechanisms against radiation through 3.8 billion years of evolutionary history, can tolerate 1000 mSv/y if radiation dose rates are low. In fact, people have lived for generations without adverse health effects in high background radiation areas such as Kelara (35 mSv/y), India, and Ramsar (260 mSv/y), Iran. Low dose radiation itself is harmless, but fear of radiation is vitally harmful.

**Conclusions:**

When people return to the evacuation zones in Fukushima now and in the future, they will be exposed to such low radiation doses as to cause no physical effects. The most threatening public health issue is the adverse effect on mental health caused by undue fear of radiation.

## Background

Soon after the Fukushima accident, people who had lived in the evacuation zone area––within a 20 km radius from the Fukushima Daiichi Nuclear Power Plant (FDNPP) of Tokyo Electric Power Company (TOPCO) ––were forced to evacuate. The International Commission on Radiological Protection (ICRP) issued an emergency recommendation on March 21, 2011 [1]. The recommendation was that reference levels for the highest planned residual dose are set in the band of 20–100 mSv. When the radiation source is under control, reference levels are in the band of 1–20 mSv/y. Therefore, 100 mSv at first and later 20 mSv could be set as the limit doses in time of great emergency. The Japanese government, however, set the limit dose as low as 1 mSv for the public in the name of safety. This low dose conversely impressed danger of radiation and stirred up fear of radiation, inducing more than 1600 accident-associated deaths, which is one of tremendous human, social, and economic losses. As some evacuees are returning to their homes now, it is of importance to learn present and future contamination levels and to evaluate their effects on physical and mental health.

For full understanding of this review, linear no-threshold model (LNT) and the lifespan study of Atomic bomb survivors (LSS) are briefly summarized at first.

### Background knowledge of linear no-threshold model (LNT)

#### Fabricated LNT without supporting data

Muller discovered that X-rays can induce mutations in *Drosophila melanogaster* [2]. Atomic bombs had been dropped on Hiroshima and Nagasaki in 1945. The consequent fear of radiation might have supported the award of a Nobel Prize to him in 1946. He had believed that even the smallest amount of radiation is hazardous to human genes. Before delivery of the Nobel lecture [3], Muller knew the existence of a threshold [4], but he asserted that there is no threshold dose. He had to defend his faked LNT with the prestige of the Nobel prize to the bitter end. Muller and his colleague, Stern, chose together to denounce reliable Capsari’s data that shows a threshold and to accept abnormal Uphoff’s data, publicly stating that the control data by Capsari were abnormally high [5]. This was the start of fabrication of LNT.

#### Deep involvement of the Rockefeller Foundation in the promotion of failed LNT

Standard Oil Co. Inc. was established by John Rockefeller in 1870. The Rockefeller Foundation (RF) was threatened by the discovery of atomic energy. In 1954, RF chose to finance six projects to evaluate atomic radiation [6]. RF asked the U.S. National Academy of Sciences (NAS) to organize the whole program, which was conducted under the auspices of Bronk, president of the Rockefeller University, president of NAS, and an RF trustee. RF’s grants to NAS amounted to $275,000. The Genetics Panel (GP), a committee of the Biological Effects of Atomic Radiation (BEAR) of NAS was established in 1954 and was chaired by Weaver, an RF officer. During 1956, RF awarded grants amounting to $991,000 in genetics, most of which were awarded to four American universities, for which Muller, Sonneborn, Glass (a student of Miller), and Crow (a colleague of Miller) worked. They were members of GP. Because most of 17 members believed that all doses of radiation were harmful, irreversible, cumulative, and linearly acting, no significant discussion occurred [7]. GP recommended LNT on June 12, 1956 [8], abandoning the threshold of 500 mGy/y since 1934. The next day, the New York Times, owned by an RF trustee, reported on LNT on the front page. Other media followed. Soon after its publication, several leading biologists asked GP to provide documentation to support the LNT. GP informed the president of NAS, Bronk, that it would not provide any documentation; right from the start, they did not have relevant data. In the long run, NAS accepted GP’s actions. Therefore, one must conclude that NAS was complicit in the falsified LNT recommendation.

### Exposure dosimetry of Atomic bomb survivors

#### Changes of dosimetry systems for four times

At first, exposure doses were estimated using data collected from Atomic bomb explosion tests on the ground in the Nevada desert. Atomic bombs dropped on Hiroshima and Nagasaki were detonated at 600 m and 503 m heights, respectively. To obtain more accurate data, the ICHIBAN project was planned, in which a 510 m high tower was constructed in the Nevada desert [9]. A nuclear reactor was placed on the top of the tower and data were collected. The dosimetry of the CHIBAN project was named tentative dose 1965 (T65D). Around the 1980s, it was found that T65D did not correctly reflect the intensity of Atomic bomb radiation. Exposure doses were reexamined and Dose System 1986 (DS86) was established [10]. In around the 1990s, DS86 was revised again and Dose System 2002 (DS02) was established [11]. DS02 is used to estimate the exposure doses of Atomic bomb survivors. Thus, T65D is the basic dosimetry system and DS86 and DS02 are modified versions.

#### Black rain never falls in the Nevada desert

The energy of a typical Atomic bomb was divided into three components: 35 % thermal radiation (heat and light), 50 % blast (pressure shock wave), and 15 % nuclear radiation (5 % prompt and 10 % residual, [12]. Of the 15, 5 % are initial radiation released within 30 s and 10 % are residual radiation, which consists of minor induced radioactivity and major fallout [13]. Black rain never falls in the Nevada desert and radiation doses were estimated by only initial radiation (5 %). At Hiroshima and Nagasaki, thermal radiation incinerated or scalded plants, animals including humans, houses, and other organic substances. From the many waterways in Hiroshima, a large volume of water was evaporated, forming part of the mushroom cloud. The vapor went up into the sky and cooled thereafter to form raindrops containing soot and other debris; the resultant black rain started to pour down 20–30 min after the detonation. Therefore there is the possibility that black rain included twice as much radiation as the initial radiation. However, accurate estimation of exposure doses from residual radiation is quite difficult in spite of long years of research and findings are disparate and inconclusive still now [14]. A report shows that the region west to the hypocenter has a higher cancer risk compared to other areas, suggesting the adverse effect of black rain [15]. Another report indicates that rain exposure shortly after the atomic bombings in Hiroshima and Nagasaki is unlikely to increase cancer risk, although deleterious health effects cannot be completely ruled out [16].

#### Evidence for underestimation of exposure doses

It is difficult to integrate residual radiation included in back rain into exposure doses, because black rains did not fall evenly, the blackness differed depending on areas and time, and information was based on testimonies of the residents. When one considers that residual radiation (10 %) constituted twice as much radiation than the initial exposure (5 %), then neglecting the effects of residual radiation made exposure doses underestimates. To what extent were the exposure doses underestimated? It must be at least by half. First, the residual radiation dose constituted twice as much radiation as initial one. Second, blood in the stool and diarrhea in cattle and deaths of fish were reported in areas, where direct effects of γ-rays and neutrons were negligible. Third, the report by the United Nations Scientific Committee on the Effects of Atomic Radiation (UNSCEAR) [17] indicates that *hibakusha* who lived 1500–1999 m from the epicenter were estimated as exposed to 500 mGy, at which no subjective symptoms were expected. Almost all cases of leukemia, however, showed severe radiation complaints that are expected to occur at doses more than 2 Gy. These data and reports strongly support that exposure doses were underestimated at least by half, and more plausibly four times.

### Does lifespan study of Atomic bomb survivors (LSS) support LNT?

#### No linear dose–response

LSS has provided fundamental data to support the NLT. The latest published result of LSS analyzed 10,929 cancer deaths out of 86,611 deaths during 1950–2003; 527 cases were attributed to exposure to radiation [18]. The authors insist that the dose response is linear without thresholds (LNT) and ERR was 0.42. At higher doses, however, people are liable to die before cancer development and downturn appears. The downturn itself contradicts a linear dose response. At mid doses, solid cancer deaths match the linear-quadratic fit (LQ) better than linearity. At low doses, no signify significant differences were seen between survivors and the control. On the contrary, hermetic effects were seen, i.e., survivors showed a lower incidence of cancer deaths. Leukemia, a cancer of the blood cells, is a better indicator of radiation than problematic solid cancers because it is sensitive to radiation. It appears 2 years after exposure and reaches a peak 6–8 years later. The relative risk per Gy of leukemia is around 4.9, whereas that of solid cancers is 1.29 [19]. The dose-response of leukemia is not linear, but the linear-quadratic or sigmoidal if higher doses are integrated. Linearity must be realized transiently in a limited phase from the linear-quadratic curve to downturn. Taken together, the inference of linear dose response is not the best choice, but rather a wrong choice.

#### Existence of threshold

Radiation doses were underestimated at least by half or cancer deaths were overestimated more than two-fold. If this tendency for underestimation is incorporated into calculations, a threshold would be set, as an earlier report noted that a threshold could be set at 0.04 Gy [20]. Although “a formal dose-threshold analysis” showed no threshold [18], the use of a different analytical method detected thresholds [21]. The LSS report describes that the dose and dose-rate effectiveness factor (DDREF) are close to unity [18]. As *hibakusha* were irradiated acutely, no dose-rate effect can reasonably be expected. On the other hand, Fukushima residents have been exposed to radiation at low dose-rates for elongated periods. Tanooka analyzed DDREF precisely and concluded that DDREF would be 16.5 [22]. Therefore, underestimation of radiation doses at least by half and contribution of DDREF provide at least 33-fold less risk of cancer deaths than that predicted from LSS [18]. Introduction of DDREF to risk estimation of low dose radiation, thresholds would be surely established in Fukushima. Hormesis is seen even in LSS as shown below.

### Adaptive responses or hormesis acquired through evolutionary history

#### Typical examples of adaptive responses or hormesis

**Fig. 1:**
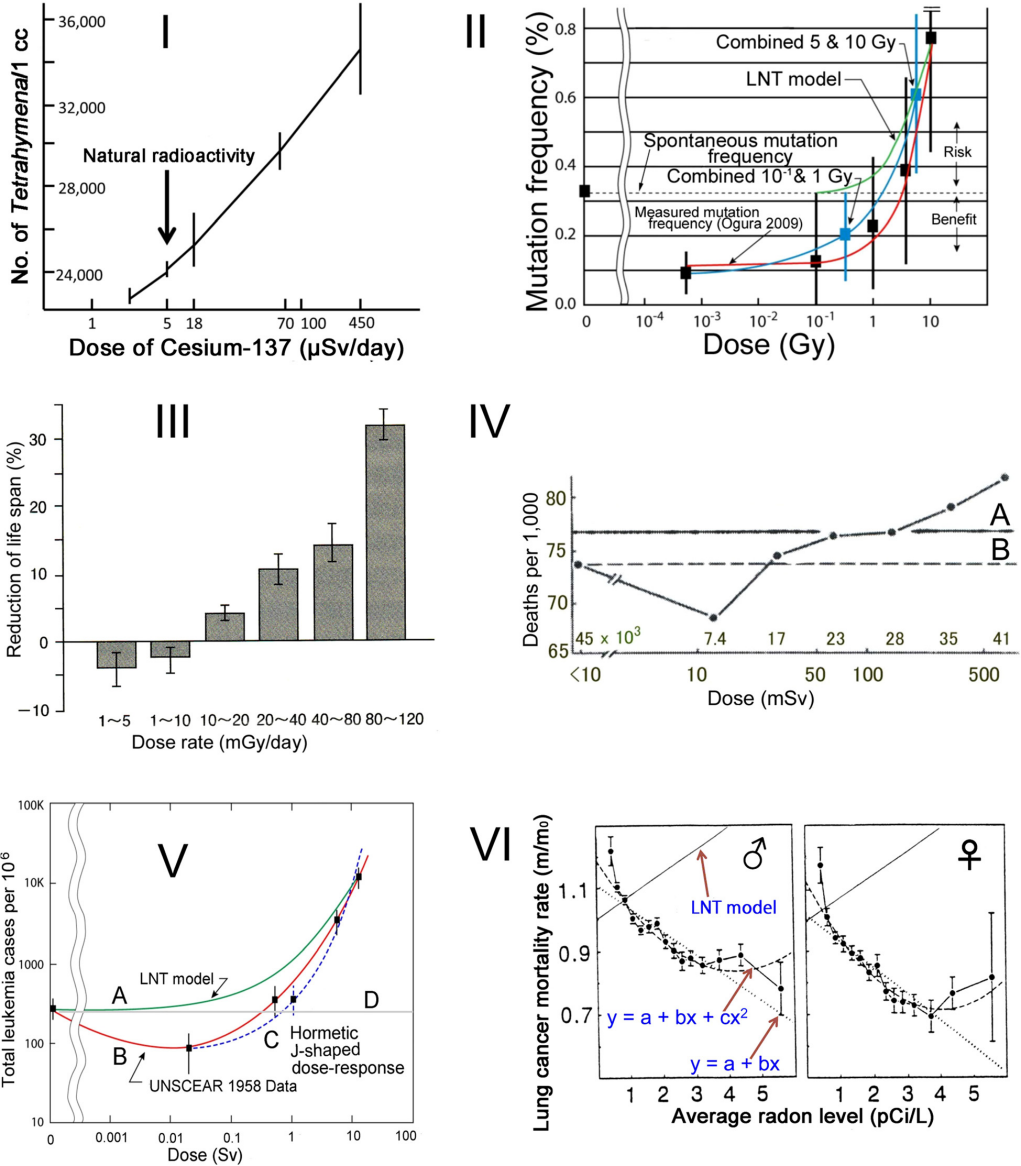
Examples of adaptive responses or hormesis. I, growth stimulation of *Tetrahymena pyriformis* by γ-rays [23]. II, hormetic effects of γ-rays on mutation induction in *Drosophila melanogaster* [29]. III, life span extension by life-ling γ-ray irradiation in mice [26]. VI, hormetic effects of radiation on solid cancer induction in Atomic bomb survivors in Hiroshima [28]. V. hormetic effects of radiation on leukemia induction in Atomic bomb survivors in Hiroshima [29]. VI, decreased lung cancer mortality in regions of high radon levels [30]

Even minimal doses of radiation are regarded as hazardous if one uses LNT as a guide. Therefore, any evidence of a beneficial response is sufficient to contradict LNT. A PubMed search yielded 33,134 hits for adaptive response and 1336 hits for hormesis as of February 28, 2016. From the large body of evidence for adaptive responses or hormesis, a few illustrative examples can be described herein (Fig. 1). Adaptive responses or hormesis are apparent throughout living organisms. *Tetrahymena pyriformis,* a protozoan, shows growth retardation under lessened radiation conditions, but showed growth enhancement proportionally to increased radiation doses [23] (Fig. 1, I). This is reminiscent of the recent finding that bacterial growth was inhibited deep underground, where radiation was reduced to one four hundredth of its level at the Earth’s surface [24]. X-rays [25] and γ-rays [26] induced mutations in *D. melanogaster* with hormesis and thresholds (Fig. 1, II). The dose–response relation was not linear, but was instead J-shaped, indicating a hormetic effect induced by 1 Gy or less. These findings clearly contradict Muller’s results and LNT. The life span of mice was extended by life-long γ-ray irradiation at 1–10 mGy/day (365–3650 Gy/y), 200–2000 times higher than the natural radiation dose [27] (Fig. 1, III). Hormetic effects are apparent in solid cancer induction in Atomic bomb survivors [28] (Fig. 1, IV). The cancer mortality rate of people in villages northwest of Hiroshima (Fig. 1, IV, A) was higher than that of “in-the-city control” from 3 to 10 km from ground zero (Fig. 1, IV, B). Leukemia incidence among the Hiroshima Atomic bomb survivors was depicted by Cuttler [29] from the original table [17] (Fig. 1, V). The LNT model does not fit the leukemia incidence (A). At 0.02 Sv, the incidence is clearly less (B) than the control (D). The data fit a J-shaped dose-response (C), suggesting hormesis. Asserting that dosimetry data were uncertain, UNSCEAR [17] did not accept this finding. Lung cancer mortality rates vs. average radon concentrations clearly indicate hormesis and contradict the LNT model (Fig. 1, VI) [30]. These examples of clearly hormetic effects decisively contradict LNT.

**Fig. 2:**
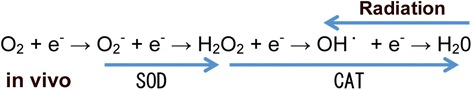
Formation of reactive oxygen species (ROS) and their elimination by enzymatic reactions. O_2_^-^, H_2_O_2_, and OH^•^ are ROS and O_2_^-^ and OH^•^ are radicals that carry an unpaired orbital electron in the outer shell. SOD is superoxide dismutase and CAT, catalase

#### Biological basis of hormesis as a homeostatic defense mechanisms

NHK TV once reported that rats captured in the Chernobyl Exclusion Zone showed neither DNA damage nor elevation of DNA repair systems, but showed an increased radical scavenger level. When birds of 16 species captured in that zone were examined, the level of glutathione, a radical scavenger, was found to be elevated [31]. The authors argue that the result reflects an aspect of hormetic effects. Animals and birds are able to erase reactive oxygen species (ROS) and radicals, major products of radiation, before these agents damage DNA.

Primary radionuclides associated with fission such as ^238^U, ^232^Th, ^235^U, and ^40^K have existed since the creation of the earth 4.6 × 10^9^ y ago. Living organisms have evolved for 3.8 billion years under exposure to radiation. It is estimated that background radiation exposure has dropped from approximately 7.0 to 1.35 mGy/y during the period of evolution on earth [32]. If living organisms could not have acquired defense mechanisms against radiation, they would not exist.

The major component of our body is water (70–80 %). The major effect of low-linear energy transfer radiation (LET) is ionization of water to form ROS and/or radicals such as OH^•^, H_2_O_2_, and O_2_^-^, which constitute major sources to damage DNA (Fig. 2). Therefore, the front line defenders are radical scavengers and antioxidants. Animals and birds in the Chernobyl Exclusion Zone are making full use of radical scavengers and antioxidants before DNA is damaged. Nrf2 plays an important role in oxidative stress response in mammalian cells by regulating the expression of a battery of far more than 100 cytoprotective genes associated with glutathione metabolism, antioxidant enzymes, drug detoxifying enzymes, and so forth. Ionizing radiation activates Nrf2 and can therefore ameliorate various oxidative stresses including radiation by restoring redox homeostasis. Nrf2 functions through the Keap1-Nrf2 stress response pathway [33].

What is the range of defense by redox homeostasis? Daily respiration produces 10^9^ ROS/cell and leaves 0.1 double strand breaks (DSB)/cell, whereas 1 mSv leaves 0.0001 DBS/cell [34]. Granted that ROS produced by daily respiration is within the range of defense capacity and that DSB incidence reflects the ratio of defensibility, the DSB ratio of 0.1 : 0.0001 indicates that 1 mSv occupies only 1/10^3^ of the defensibility. In other words, 1 Sv is defensible. LSS data show that 0.4–0.6 Sv are hormetic. When radiation doses were underestimated by half, the doses were actually 0.8–1.2 Sv. Figure 1, II shows that 1 Sv is a threshold. Doses of 365–3650 Gy (Sv)/y elongated life span of mice (Fig. 1, III). Around 1 Sv is the threshold for leukemia induction by an Atomic bomb (Fig. 1, V).

Radiation damages DNA directly and indirectly. Most types of LET such as background radiation and that of Fukushima areas act indirectly by ionizing water of our body to form ROS, which are readily quenched by redox homeostasis mechanisms. Some leaked ROS induce DNA damage, most of which are repaired. People with defects in repair systems are prone to cancer. DNA-damaged cells stop cell division and wait for the completion of repair. When cells fail to repair the damage, they die off by apoptosis. Tough cells might manage to pass through these defense mechanisms and become cancer cells, but the immune systems are waiting to eliminate them. People with defects in the immune systems are prone to cancer. These various defense mechanisms must serve as the basis for adaptive response or hormesis. Failure of LNT resides in the neglect of these biological systems that have been acquired during 3.8 billion years of evolutional history.

#### Theory to explain thresholds by homeostatic defense mechanisms

**Fig. 3:**
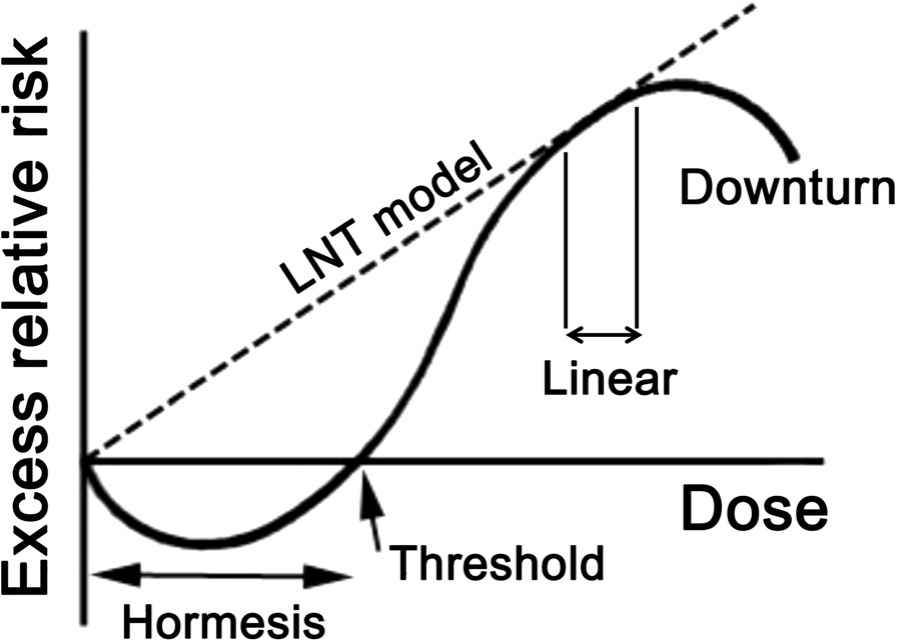
Schematic illustration of the relation between LNT, thresholds, and hormesis at chronic irradiation. When DDREF = 1 by acute and intensive irradiation, dose–response approximates a linear line in a limited dose range at higher doses. Even with acute irradiation, radiation effects of lower doses are nullified by powerful and efficient defense mechanisms in which adaptive response induces hormesis. When DDREF > 1 by chronic and mild irradiation, low and mild doses are under a range of adaptive response and hormesis occurs. The limit of the defense capacity determines a threshold. At high doses beyond the threshold, adverse effects appear. A linear response (*dashed line*) is realized transiently in a limited dose range immediately before the downturn

Because LNT assumes an excess risk of cancers from even the smallest amount of radiation exposure, dose responses follow only a rising straight line from bottom left to top right (Fig. 3, *dashed line*) and never occur below the bottom line. It follows then that responses under the bottom line demonstrate a failure of LNT. In this sense, each plate in Fig. 1 depicts that failure of LNT. Figure 3 schematically depicts why thresholds appear. Main effects of low to mid-dose radiation are production of ROS and radicals, which are protected by efficient defense mechanisms. The fundamental nature of living organisms is incessant response to stimuli, one of which is radiation that induces adaptive response or hormesis. Dynamic responses are reminiscent of Paracelsus’ aphorism that the dose makes the poison. Lower doses of radiation are actually beneficial: higher doses are hazardous.

### Unnecessary evacuations: misgovernment has been driving Fukushima people to death, victims of the falsified LNT Dogma

#### Lessons of Chernobyl and thyroid cancer cases in Fukushima

The Chernobyl accident was the most severe in the history of the world nuclear industry. Still, direct radiation effects have been marginal. The 116,000 evacuees did not die of radiation as was the case of Fukushima. However, of 134 firefighters engaged in fire extinguishing activities, 28 died because of radiation sickness and subsequent diseases during the first 4 months [35]. They must be considered to have been murdered by occupational orders rather than by radiation. The other 106 people were recovered, but 19 of them died during the next 20 years. These 19 might not be victims of radiation because the spontaneous annual death rate is about 1 %: 106 × 20 × 0.01 = 21.2 > 19. In highly contaminated areas of the former Soviet Union, there have been no reliable reports to show increases of solid cancers or leukemia. Thyroid cancer is the main health concern. During 1991–2005, more than 6000 cases were reported, of which most were attributable to drinking milk contaminated with ^131^I, and 15 persons died up to 2005 [35]. Most thyroid cancers are benign papillary thyroid cancer; if they had been malignant cancer, 2000–3000 persons would have died. The thyroid is a radiation-insensitive organ. Its latent period is not fixed, but seems to be around 10 years or more.

An approximately 30-fold increase in the number of thyroid cancer cases among children and adolescents less than 18 years in Fukushima was reported recently [36]. They found 110 cases (0.00368 %) among 298,577 examinees (81 %) out of 367,687 candidates. Fukushima Prefecture was divided into three areas, i.e., the most contaminated area (subarea 1), the moderately contaminated area (subareas 2–5), and the least contaminated area (subareas 6–9) and cancer incidents were 0.00195, 0.00401, and 0.00272 %, respectively. Their analyses is, however, difficult to understand how to reach 30-fold increase, because the subarea 7 was chosen as the reference and its prevalence odds ratio (POR) is 1 and its incidence rate ratio (IRR) is 20, while POR’s other areas are 1.3–2.6 and IRR’s are 25–50. There seems to be no big differences without respect to contamination levels. This fear-mongering article was refuted more recently [37]. Before and after the Fukushima accident, average cancer sizes were significantly different (4.1 vs 1.4 cm). Large-scale and sophisticated screening might allow identifying many thyroid cancers among Fukushima’s children and adolescents. Thyroid cancer patients’ average age at surgery was also older among the post-Fukushima accident patients (age 17.4 vs 11.9 years), implying that cancer had started to develop prior to radiation exposure. In any rate, there are no substantial grounds for accepting that the cancer cases are due to the nuclear accident.

Newspapers reported on October 21, 2015, that a leukemia-stricken welder who worked in the FDNPP from November 2011 to December 2013 and exposed to 15.7 mSv won compensation from the government for the first time, and some media suggested that low dose radiation could induce cancer. Since 1976, the public exposure limit was 5 mSv/y and any men who had been exposed to more than 5 mSv/y and contracted cancer after 1 year or later could be qualified for workman’s compensation. Judging form the exposure dose and elapsed time, the leukemia is quite unlikely to be attributable to the nuclear accident.

#### Contamination levels in Fukushima

Officially, WHO and UNSCEAR have predicted that cancers in Fukushima will not increase. In contrast, the severe social and economic depression of the affected areas and the associated grievous psychological problems of the general public and emergency workers have taken a heavy toll [38]. The same is true for Fukushima: evacuation and long-term displacement created severe health-care problems especially for aged residents [39]. There have been no victims of radiation itself in Fukushima. Abundant wildlife populations in the Chernobyl Exclusion Zone have made it a magnificent nature preserve [40]. Although abnormalities of butterflies captured around Fukushima areas have been reported [41], they must not be caused by genetic mutation [42].

ICRP’ recommendation on March 21, 2011 [1] was that reference levels could be set in the band of 20–100 mSv at fist and when the radiation source is under control, reference levels be in the band of 1–20 mSv/y. Actual exposure dose levels should be examined. UNSCEAR estimated that the people in Okuma Town would be exposed to the maximal radiation dose in Fukushima, 4.9 mSv, during 1 year when evacuees return to their homes on March 1, 2014 [43]. Examination of external doses of 421,394 residents for the first 4 months after the accident was 62.0 %, <1 mSv; 94.0 %, <2 mSv; 99.4 %, <3 mSv [44]. Other estimated doses in three areas of Fukushima were 0.6–2.3 mSv/y in Tamura City, 1.1–5.5 mSv/y in Kawauchi Village, and 3.8–17 mSv/y in Iitate Village [45]. These data indicate that no evacuation was needed in Fukushima. The then government of the Democratic Party of Japan chose, of all of the range of choices available, the minimum dose of 1 mSv as the limit dose for the public, neglecting ICRP’s recommendation during the time of emergency. This decision induced tremendous human, social, and economic losses. The mortality risk of residents in nursing homes evacuated after the Fukushima accident was 2.7-fold higher than those who remained there before the accident [46], which indicates that the stress of evacuation life is much riskier than that of radiation exposure. Even disregarding nuclear accidents, people are known to live healthy active lives without any measurable adverse health effects in areas with much higher natural background radiation than the prevailing radiation levels in Fukushima [47].

## Discussion

The author had been taught that even the smallest amount of radiation is dangerous and believed the LNT as dogma until undertaking volunteer activities in Fukushima [48, 49]. On the occasion of the Fukushima accident, the author found after intensive study that low dose radiation is not hazardous but beneficial [50]. Before writing this review, a Japanese edition was written from a wider viewpoint [51]. A body of evidence refuting the fabricated LNT has been accumulated. Nevertheless, LNT recommended in 1956 by the NAS, the highest authority in the scientific world, has been rigidly integrated into establishments such as governmental, academic, and other systems, and even into our central nervous systems makes it difficult to overturn LNT. Actually, RERF, a Japan–US joint organization, conducted LSS and has published results insisting that LNT is correct [18, 20]. Using LSS data, BEIR, a committee of NAS, has published influential reports, one of which is BEIR VII-Phase 2, which strongly advocates that LNT is correct [52]. RERF and BEIR data were used in influential UNSCEAR reports that formed the basis of ICRP recommendations [53], which became, in turn, the basis for regulatory guidelines worldwide. Most major media stir visceral fear into an image of danger associated with radiation and neglect the beneficial aspects of radiation. Risky evacuation was undertaken in the name of safety, but in fact evacuation itself was dangerous and has been driving vulnerable people to death. As a measure of self-protection, the author presents a message of accurate radiation information to encourage the public, especially the people of Fukushima who have returned or who are expected to return home, to reject fear of radiation caused by fabricated LNT.

## Conclusions

1. According to the LNT hypothesis, even the smallest amount of radiation is hazardous. In fact, LNT is not based on solid data and is a product of fabrication.
2. The LSS provided basic data for LNT. Dose estimation of LSS was underestimated and overestimated cancer risk accordingly. LSS does not support LNT any longer.
3. Living organisms have established potent and efficient defense mechanisms against radiation through their evolutional history of 3.8 billion years. Results show that adaptive response is manifested as hormesis: low-radiation to mid-radiation doses are beneficial, although high doses are hazardous.
4. When people return to the evacuation zones in Fukushima now and in the future, they will be exposed typically to around 5 mSv (ca. a medical diagnosis dose) or less. Actually, many people live in areas with much higher natural background radiation without adverse health effects. No health problems are expected to occur after their return home.
5. At the dose levels in Fukushima, radiation *per se* is expected to cause no physical effects. The most threatening and debilitating public health issue is the adverse effect on mental health caused by undue fear of radiation.
6. An urgent task for government is to release people from the spell of LNT and to abandon LNT to establish a new radiation protection paradigm based on correct scientific knowledge, including radiation hormesis.
